# Effectiveness of an Oral Supplementation of Phycocyanin and Palmitoylethanolamide for a Short-Term Prophylaxis of Menstrual Migraine: A Retrospective Observational Study

**DOI:** 10.3390/biomedicines14040865

**Published:** 2026-04-10

**Authors:** Gianni Allais, Massimo Autunno, Florindo D’Onofrio, Luisa Fofi, Maria Gabriella Saracco, Fabiola Bergandi, Chiara Benedetto, Francesca Silvagno, Loredana Bergandi

**Affiliations:** 1 Department of Surgical Sciences, Women’s Headache Center, University of Turin, 10126 Turin, Italy; gb.allais@tiscali.it (G.A.); fabiola.bergandi@gmail.com (F.B.); chiara.benedetto@unito.it (C.B.); 2 Department of Clinical and Experimental Medicine, University of Messina, 98125 Messina, Italy; mautunno@unime.it (M.A.); 3 Neurology Unit, San Giuseppe Moscati Hospital, 83100 Avellino, Italy; florindo.donofrio@libero.it (F.D.); 4 Headache and Neurosonology Unit, Fondazione Policlinico Campus Bio-Medico, 00128 Roma, Italy; l.fofi@policlinicocampus.it (L.F.); 5 “Affidea” Headache Center, 14100 Asti, Italy; saraccomgg@gmail.com (M.G.S.); 6 Department of Oncology, University of Turin, Via Santena 5 Bis, 10126 Turin, Italy; francesca.silvagno@unito.it (F.S.); loredana.bergandi@unito.it (L.B.)

**Keywords:** menstrual migraine, phycocyanin, palmitoylethanolamide, short-term prophylaxis

## Abstract

**Background**: Menstrual migraine (MM), including pure menstrual migraine (PMM) and menstrually related migraine (MRM), is characterized by attacks occurring in close temporal association with menstruation and is often more severe, longer lasting, and less responsive to treatment than non-menstrual migraine. Prostaglandin-mediated inflammation and calcitonin gene-related peptide (CGRP) release play a key role in MM pathophysiology. Phycocyanin (PC) and palmitoylethanolamide (PEA) are nutraceutical compounds with anti-inflammatory, analgesic, and neuroprotective properties that may be beneficial as short-term perimenstrual prophylaxis. **Objectives**: To evaluate the effectiveness of an oral supplementation combining phycocyanin and palmitoylethanolamide as a short-term prophylaxis for menstrual migraine in a real-world clinical setting, a retrospective observational study without a control group was conducted in five Italian centers between May 2023 and June 2025. **Methods**: Clinical records of 800 women were reviewed, and 220 patients receiving perimenstrual supplementation with phycocyanin and palmitoylethanolamide were screened. Sixty-one women diagnosed with migraine without aura, according to the International Classification of Headache Disorders, met all inclusion criteria and were analyzed. Phycocyanin and palmitoylethanolamide were taken at a dosage of two capsules daily from five days before to five days after the onset of menstruation for three consecutive months. Outcomes during the perimenstrual window were compared with a three-month period without supplementation. Primary outcomes included migraine severity, frequency, and duration of the attacks; secondary outcomes included analgesic consumption and menstrual migraine-associated symptoms. **Results**: Among the 61 included patients, phycocyanin and palmitoylethanolamide supplementation was associated with a significant reduction in migraine severity across all monitored perimenstrual days (*p* < 0.0001). While the overall monthly frequency of migraine attacks did not change, the number of migraine days during the perimenstrual window significantly decreased from the first month of supplementation (*p* < 0.05). Moreover, migraine duration during the perimenstrual window was significantly reduced at one, two, and three months of phycocyanin and palmitoylethanolamide supplementation compared with baseline. Analgesic use and the number of days with migraine-associated symptoms (nausea, vomiting, photophobia/phonophobia) were also significantly reduced. Treatment was well tolerated. **Conclusions**: In this real-world retrospective study, perimenstrual supplementation with phycocyanin and palmitoylethanolamide was associated with reduced severity, duration, and perimenstrual frequency of menstrual migraine attacks, along with decreased analgesic use, suggesting a safe and potentially beneficial short-term prophylactic strategy for women with menstrual migraine.

## 1. Introduction

Menstrual migraine (MM) is a special type of migraine associated with the ovarian cycle [1]. The primary distinguishing feature between menstrual and non-menstrual migraines is the timing of the attacks in relation to the menstrual cycle and their strong association with hormonal fluctuations, especially the drop in estrogen levels that typically occurs just before menstruation begins [2]. The symptoms of menstrual migraine are similar to those of general migraines, including unilateral or bilateral throbbing pain, nausea, vomiting, photophobia and phonophobia, whereas the intensity and frequency of migraines during menstruation are often greater compared to non-menstrual migraines. Indeed, menstrual-associated migraines tend to be more severe, last longer, and be less responsive to treatment than migraines occurring at other times in the cycle [3].

According to the International Classification of Headache Disorders, it is classified into two main categories: pure menstrual migraine (PMM) and menstrually related migraine (MRM) [4]. PMM, affecting about 10–20% of women with migraine, refers to migraines that occur exclusively during perimenstruation days and that are triggered by the decline in estrogen levels [5] and the associated changes in neurotransmitter function [6], consistently occurring from 2 days before to 3 days after the onset of bleeding without any migraine attacks occurring at other times of the month [1]. Usually, the treatments of PMM are symptomatic (triptans, nonsteroidal anti-inflammatory drugs (NSAIDs) and antiemetics) even if it is common to use a short-term prophylaxis on the days surrounding menstruation with both pharmacological or non-pharmagological products [7]. MRM, affecting about 60–70% of women with migraine, refers to migraine attacks that are triggered by hormonal fluctuations during the two days before and the three days after the onset of menstruation, primarily related to estrogen withdrawal and other hormonal fluctuations just before menstruation, but also in other days of the cycle with no correlation to hormonal fluctuations. Treatment options for MRM include acute medications to relieve symptoms but also many preventive drugs currently use for migraine prophylaxis, recently including a calcitonin gene-related peptide (CGRP) receptor antagonist or CGRP monoclonal antibodies, too [8].

In addition to pharmacological treatments, several non-pharmacological strategies, among which are cognitive behavioral therapy [9], acupuncture and acupressure [10], lifestyle modifications and regular magnesium supplementation [11], particularly during the premenstrual phase, have been shown to manage both PMM and MRM, reducing the frequency of migraine attacks.

The decline in estrogen levels around menstruation plays a pivotal role in triggering menstrual migraines influencing the occurrence and severity of migraines [12]. One of the key mechanisms through which the estrogen fall influences migraine development is by upregulating cyclooxygenase (COX) enzymes, particularly COX-2 [13]. This enzyme is involved in the production of prostaglandin-2 (PGE2), which is an inflammatory molecule [14] released from cell membranes during an inflammatory response [15]. The role of prostaglandins in migraine pathophysiology is still under investigation; however, several mechanisms have already been identified [16]. Prostaglandins contribute to increased blood flow and vascular inflammation, neuronal excitability, and sensitization to pain [17,18], which are key features of migraine attacks.

Migraine is thought to be triggered by multiple neurovascular mechanisms [19], including activation of the trigeminovascular system and neurogenic inflammation through the release of vasoactive neuropeptides like nitric oxide (NO) and the CGRP involved in vasodilation and migraine pain [20]. Indeed, neurogenic inflammation in the meningeal blood vessels surrounding the brain is considered a critical factor in eliciting migraine pain [21]. This process, involving vasodilation and peripheral nerve sensitization, contributes not only to the onset, but also to the persistence of migraine attacks [21]. In women with migraine susceptibility, elevated prostaglandin levels, particularly during the late luteal and early menstrual phases, are also implicated in menstrual cramps and uterine pain [12] through the release of inflammatory cytokines [5]. The reduced estrogen levels lead to the decreased availability of serotonin, dopamine, and endorphins, along with a dysregulation of NO which facilitates CGRP release. This impairs the modulation and anti-inflammatory mechanisms of brain-endogenous pain, further exacerbating susceptibility to migraine [22]. It has been reported that PGE2 can enhance CGRP release from rat sensory trimeginal neurons [23] and the functional human data strongly suggest a pathophysiological link, supported by in vitro and animal studies of PGE2–CGRP interactions.

As prostaglandins contribute to migraine development, particularly by promoting inflammation and sensitizing pain pathways, anti-inflammatory treatments have been developed to target the COX enzymes responsible for their synthesis, serving as a symptomatic treatment option [24]. Indeed, NSAIDs, such as naproxen, ibuprofen, and mefenamic acid, are commonly used in both acute and short-term preventive treatment of menstrual migraine [25]. By inhibiting COX activity, they reduce prostaglandins production, thereby decreasing inflammation, vascular changes and pain associated with migraine attacks [18,26]. NSAIDs are especially effective for mild to moderate migraine episodes and may also help alleviate accompanying symptoms such as nausea and vomiting [27]. Moreover, selective COX-2 inhibitors, such as celecoxib, can specifically target the prostaglandin-mediated inflammation, and therefore can be effective in reducing the severity and duration of menstrually related migraines [28].

In this scenario, however, despite their efficacy, NSAIDs and COX-inhibitors were associated with significant adverse events when administered in long-term therapies [29]. Phytotherapy represents a promising alternative with beneficial properties and minimal side effects [30] in the management of menstrual migraine, particularly as a short-term approach during the perimenstrual window (PMW) [31]. Among the wide range of available phytotherapy compounds, two emerging molecules are represented by phycocyanin (PC) and palmitoylethanolamide (PEA). PC is a protein from the Cyanobacteria with antioxidant and anti-inflammatory properties [32] and PEA exhibits pain-relieving and neuroprotective properties [33]. As both compounds show potential for migraine management by targeting inflammation, alleviating pain, and providing neuroprotection, the aim of this study was to determine the effectiveness of an oral compound consisting of a combination of phycocyanin and palmitoylethanolamide in reducing the severity, frequency, and duration of migraine attacks in women diagnosed with menstrual migraine.

## 2. Materials and Methods

### 2.1. Subjects and Study Design

This retrospective observational study without a control group was designed and carried out with the approval of the *Città della Salute e della Scienza* Ethical Committee (prot. nr. 0065659, 26 May 2023), covering the period from May 2023 to November 2025. It was conducted according to the last update of the Helsinki declaration [34]. All data used for the study were extracted from anonymized existing clinical records and patient-reported documentation. The requirement for informed consent was waived as this study was conducted retrospectively using exclusively fully anonymized data, and no identifiable patient information was accessed.

Clinical data from 800 adult Caucasian women aged 18–49 years were reviewed, and 220 patients receiving perimenstrual supplementation with phycocyanin and palmitoylethanolamide were screened.

The exclusion criteria were as follows: patients with chronic migraine (more than 15 migraine days per month), migraine with aura, tension-type headache, or cluster headache (International Headache Society criteria [4]), pregnant women or those planning pregnancy, patients with known cardiovascular disease, individuals non-compliant with diary completion, those using contraceptives, with irregular menstrual cycles, or recent changes in migraine prophylactic therapy- and subjects unable to swallow capsules or who discontinued treatment due to side effects.

During data extraction, records were also excluded if patients refused to complete standardized questionnaires or had a diagnosis of probable migraine. Only records documenting a diagnosis of menstrual migraine without aura—classified according to the International Classification of Headache Disorders, 3rd edition (ICHD-3) [4]—were included for data analysis, ensuring a homogeneous and well-defined study population. Specifically, data from patients with menstrually related migraine (MRM, A 1.1.2) or pure menstrual migraine (PMM, A 1.1.1) were included.

**Figure 1:**
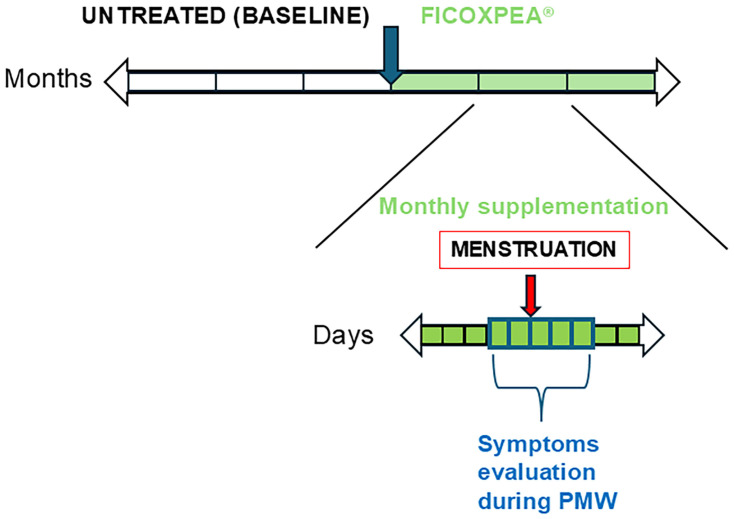
FICOXPEA^®^ supplementation schedule.

Data concerning 61 patients diagnosed with migraine without aura, and with documented supplementation of FICOXPEA^®^ were extracted. The oral dietary supplement, FICOXPEA^®^, containing 250 mg phycocyanin (Spirulina platensis extract), 200 mg PEA, and 55 mcg selenium (Kura srl, Turin, Italy), 2 capsules once daily for three months, was taken from 5 days before to 5 days after the onset of menstruation. The FICOXPEA^®^ supplementation schedule is shown in Figure 1. For these patients, a three-month observation period during which patients had recorded the natural course of their migraine attacks before the supplementation with FICOXPEA^®^ generated data that were used to document baseline patterns without any intervention from the investigators, consistent with the observational design. Records also indicated that patients used rescue medications, NSAIDs at standard doses, analgesics, triptans, or antiemetics, on an as-needed basis. No modifications to existing migraine prophylactic therapies were allowed in the six months preceding the recorded supplementation period. Outcomes of supplementation during the PMW were compared with a three-month period without supplementation. Extracted data included structured interviews previously conducted by trained healthcare professionals.

These interviews had documented demographic variables (age, educational level, occupation), clinical history (comorbidities, menstrual cycle regularity, hormonal contraceptive use, family history of migraine), migraine characteristics, age at onset, chronicity, previous and current pharmacological treatments, and potential medication overuse. Moreover, patient diaries, available in the medical files, were also evaluated. These diaries contained daily entries from two days before to three days after menstruation onset, reporting migraine episodes, severity, duration, analgesic use, and associated symptoms such as nausea, vomiting, photophobia, and phonophobia. These entries were used as observational data to assess migraine outcomes during the perimenstrual window. All data were anonymized and handled in accordance with privacy and confidentiality regulations. The analysis focused solely on evaluating real-world clinical outcomes based on extracted observational data, including migraine frequency, intensity, duration, and the use of rescue medications.

### 2.2. Variables Assessed

The study assessed both primary and secondary outcomes during PMW, defined as days −2 to +3 relative to the onset of menstruation, to evaluate the effectiveness and the impact of a FICOXPEA^®^ supplementation on menstrual migraines.

The primary outcomes included the migraine severity, frequency and duration of attacks during PMW. The presence of headache and the level of pain were measured on a fully validated [35] four-point scale where participants rated the intensity of their migraine attacks on a scale from 0 (no pain) to 3 (severe headache). These ratings were collected before and after FICOXPEA^®^ supplementation to determine changes in the presence of headache and the perception of headache pain. In addition, the frequency of migraine attacks was recorded by tracking the number of days experienced both per month and within PMW where each attack is a distinct migraine event if it is separated by at least 24 h of no symptoms. The duration of migraine attacks was documented in hours, measured from the onset of symptoms to the time of relief. These values were collected before and after supplementation to identify any differences in the reduction in attack severity, frequency and duration following a FICOXPEA^®^ supportive treatment compared to the symptoms relative to the months without FICOXPEA^®^ supplementation.

The secondary outcomes included the number of analgesics taken during the PMW and the number of days marked by the following symptoms: nausea, vomiting, or photophobia/phonophobia, during PMW.

### 2.3. Statistical Analysis

The Shapiro–Wilk test was used to assess normality. Intra-group differences between non-supplemented and supplemented patients were analyzed with paired *t*-tests for normally distributed variables, using GraphPad Prism 9.0 (GraphPad Software, Inc., San Diego, CA, USA). For the analysis of migraine attack frequency and duration, a three-level repeated measures ANOVA with Bonferroni post hoc correction was first performed to assess any differences among the three untreated time points. Since no significant differences were found for untreated groups, the mean of the untreated time points was used as the baseline for paired *t*-tests. Effect sizes were calculated using Cohen’s d for pairwise comparisons between the baseline and each of the three treated time points. Missing data were assumed to be missing at random and handled in the analysis. *p* values < 0.05 were considered significant.

Data are shown as mean ± SEM or ± 95% confidence intervals. SEM reflects the precision of the observations, not individual variability, and the CI provides the magnitude and precision of findings.

## 3. Results

**Figure 2:**
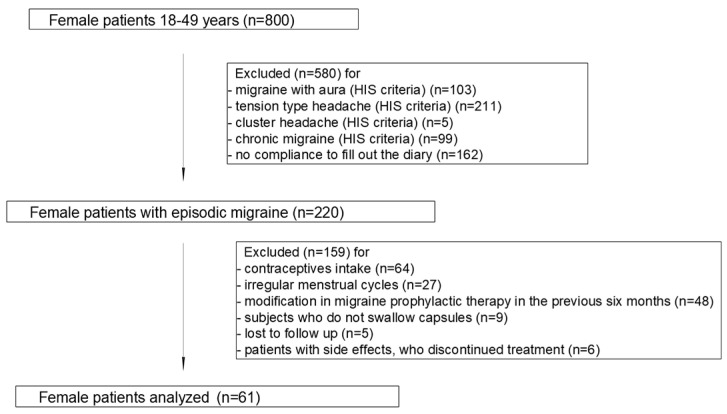
Flowchart of participant selection for the analysis.

Globally, 800 patients presented to the centers, and 220 patients with documented use of FICOXPEA^®^ were screened. Sixty-one women diagnosed with migraine without aura met all inclusion criteria and were analyzed. The trial flow diagram is shown in Figure 2.

This initial screening was conducted using more restrictive criteria than those proposed by the International Headache Society [4] (which require the presence of migraine attacks in the perimenstrual window in at least two out of three months), with the aim of selecting a population whose migraine attacks were consistently linked to the perimenstrual period. In addition, the clinical characteristics of migraine attacks were thoroughly assessed, and only women presenting attacks classifiable as migraine without aura were included; all patients who had experienced, even once during the three-month observation period, attacks classifiable as migraine with aura or probable migraine were excluded. Accordingly, only patients who, during the three-month observation period, consistently experienced attacks within the PMW were included in order to obtain a more strictly selected population clearly and consistently affected by menstrually related migraine. This approach does not conflict with the International Headache Society definition; rather, it aligns with it while applying more stringent and specific selection criteria. Furthermore, all patients using hormonal contraception and those with natural menstrual cycles shorter than 24 days or longer than 32 days were excluded. Side effects of the treatment were present in a very low number of treated patients and consisted of gastric pain (n = 4) and increase in menstrual bleeding (n = 2). FICOXPEA^®^ capsules were generally well-tolerated, and no serious adverse reactions were reported. These six women therefore discontinued the treatment early and were not included in the analysis of the study. Compliance with daily dosing of capsules exceeded 89% in participants who completed the final follow-up.

Among the 61 patients selected for this study the prevalence was 14.7% (nine women) for PMM and 85% (52 women) for MRM. Participants had a mean age of 35.7 ± 8.3 years with an average age of menstrual migraine onset of 18.3 ± 6.9 years. The distribution of occupations, comprising office workers, teachers, and hospital employees, was similar across the enrolled women.

**Figure 3:**
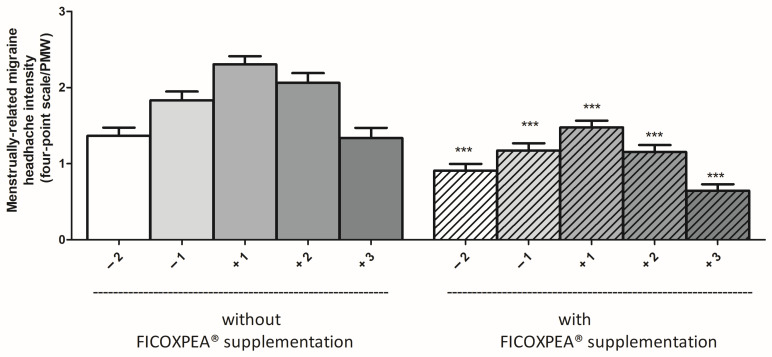
The migraine severity was recorded, during the PMW, in the period spanning from 2 days before to 3 days after the onset of menstruation, by all enrolled patients. Severity scores were averaged over a three-month period of monitoring with and without FICOXPEA^®^ supplementation, using a patient-reported four-point headache intensity scale. Mean values for each day of PMW (−2, −1, +1, +2, +3) are shown ± SEM (n = 183, <11% of observations missing at random). Statistical comparisons were performed using a paired *t*-test. *** *p* < 0.0001 compared to the same day without FICOXPEA^®^ supplementation.

Throughout all three months of the FICOXPEA^®^ supplementation phase, migraine severity was significantly reduced on each day monitored during the PMW (Figure 3).

Compared with non-supplemented, the migraine severity decreased significantly at all investigated time points: day −2 (mean difference = −0.47, 95% CI −0.66 to −0.28, *p* < 0.001), day −1 (mean difference = −0.58, 95% CI −0.77 to −0.4, *p* < 0.001), day +1 (mean difference = −0.58, 95% CI −0.92 to −0.56, *p* < 0.001), day +2 (mean difference = −0.74, 95% CI −1.04 to −0.6, *p* < 0.001), and day +3 (mean difference = −0.83, 95% CI −0.78 to −0.4, *p* < 0.001). Standardized effect sizes compared to non-supplemented were: −0.37 at day −2, −0.45 at day −1, −0.59 at day +1, −0.57 at day +2 and −0.50 at day +3.

**Figure 4:**
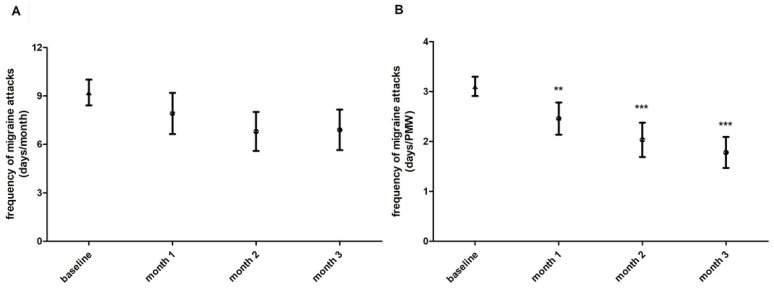
The frequency of migraine attacks, expressed as number of days per month (**A**) and during the PMW (**B**), is represented as an average over a three-month period of monitoring without FICOXPEA^®^ supplementation (baseline) and as a monthly average during the intervention period with FICOXPEA^®^ supplementation (n = 61 and n = 59, (**A**,**B**) data statistical analysis, respectively). Baseline represents the aggregated mean of three pre-supplementation month observations as the three baseline months were not significantly different and were considered to represent the same pre-supplementation condition in the graph. The data represent the means ± 95% CI. ** *p* < 0.001 and *** *p* < 0.001 compared to baseline.

Investigating the frequency of migraine attacks, the average of migraine days per month remained unchanged when comparing data obtained during each month of FICOXPEA^®^ supplementation with those without FICOXPEA^®^ supplementation, represented as baseline (Figure 4A). Notably, the number of days marked by migraine episodes during the PMW showed a significant reduction, already from the first month after FICOXPEA^®^ supplementation if compared with baseline (Figure 4B). Compared with baseline, the outcome decreased significantly at all investigated time points: month 1 (mean difference = −1.36, 95% CI −2.85 to −0.12, *p* < 0.001), month 2 (mean difference = −2.43, 95% CI −3.91 to −0.94, *p* < 0.001) and month 3 (mean difference = −2.33, 95% CI −3.81 to −0.84, *p* < 0.001). Standardized effect sizes compared to baseline were: −0.62 at month 1, −0.75 at month 2, and −0.98 at month 3.

**Figure 5:**
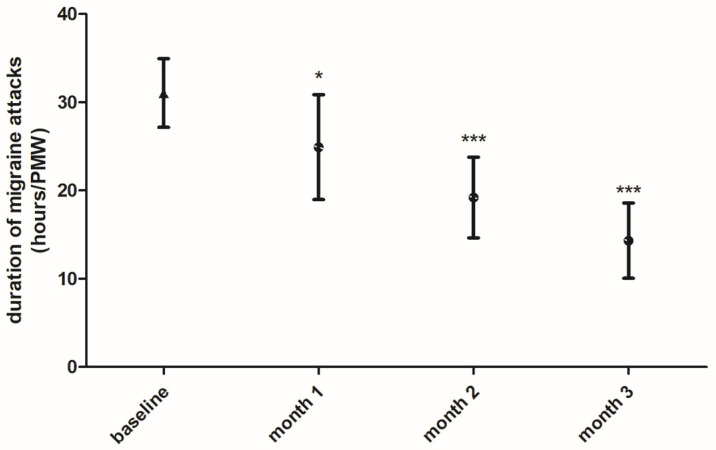
The duration of migraine attacks, expressed in hours during the PMW, is represented as an average over a three-month period of monitoring without FICOXPEA^®^ supplementation (baseline) and as a monthly average during the intervention period with FICOXPEA^®^ supplementation (n = 61). Baseline represents the aggregated mean of three pre-supplementation month observations as the three baseline months were not significantly different and were considered to represent the same pre-supplementation condition in the graph. The data represent the means ± 95% CI. * *p* < 0.05 and *** *p* < 0.001 compared to baseline.

Additionally, the duration of migraine attacks during the PMW showed a significant reduction from an average of 31.05 ± 3.4 h at baseline to 24.9 ± 2.9 (*p* < 0.05), 19.2 ± 2.3 (*p* < 0.001) and 14.31 ± 2.1 (*p* < 0.001) hours, respectively, at one month, two and three months after FICOXPEA^®^ supplementation compared to baseline (Figure 5). Compared with baseline, the outcome decreased significantly at all investigated time points: month 1 (mean difference = −6.17, 95% CI −11.5 to −0.73, *p* < 0.05), month 2 (mean difference = −12.48, 95% CI −17.9 to −7.05, *p* < 0.001) and month 3 (mean difference = −16.9, 95% CI −22.4 to −11.6, *p* < 0.001). Standardized effect sizes compared to baseline were: −0.53 at month 1, −0.56 at month 2, and −0.74 at month 3.

**Figure 6:**
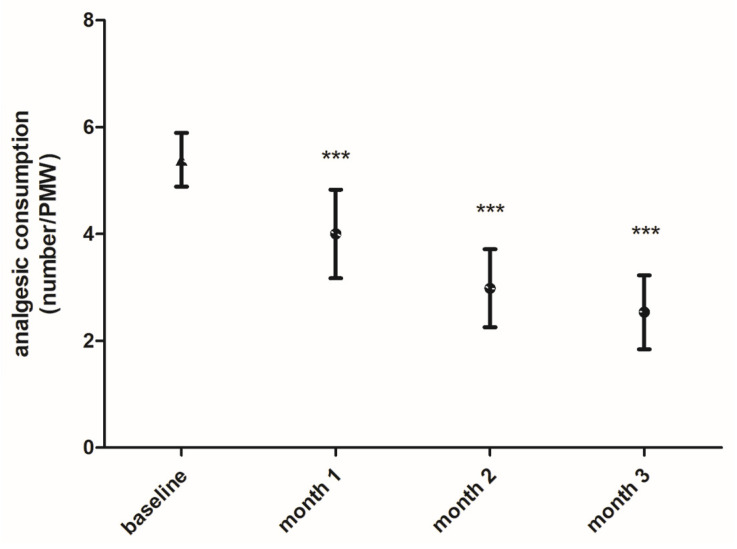
The analgesic consumption, expressed as number of analgesics taken during the PMW, is shown. Values are presented as an average over a three-month period of monitoring without FICOXPEA^®^ supplementation (baseline) and as a monthly average during the intervention period with FICOXPEA^®^ supplementation (n = 61). Baseline represents the aggregated mean of three pre-supplementation month observations as the three baseline months were not significantly different and were considered to represent the same pre-supplementation condition in the graph. The data represent the means ± 95% CI. *** *p* < 0.001 compared to baseline.

The number of analgesics taken, including NSAIDs or triptans, to treat menstrual migraine attacks was analyzed. Results revealed that analgesic consumption during the PMW was significantly reduced during the FICOXPEA^®^ supplementation phase. The effect was observed as early as the first month and continued significantly throughout the supplementation period if compared to baseline measurements (Figure 6). Compared with baseline, the outcome decreased significantly at all investigated time points: month 1 (mean difference = −1.34, 95% CI −0.63 to −0.25, *p* < 0.001), month 2 (mean difference = −2.34, 95% CI −3.05 to −1.63, *p* < 0.001) and month 3 (mean difference = −2.84, 95% CI −3.55 to −2.13, *p* < 0.001). Standardized effect sizes compared to baseline were: −0.72 at month 1, −0.95 at month 2, and −0.93 at month 3.

**Figure 7:**
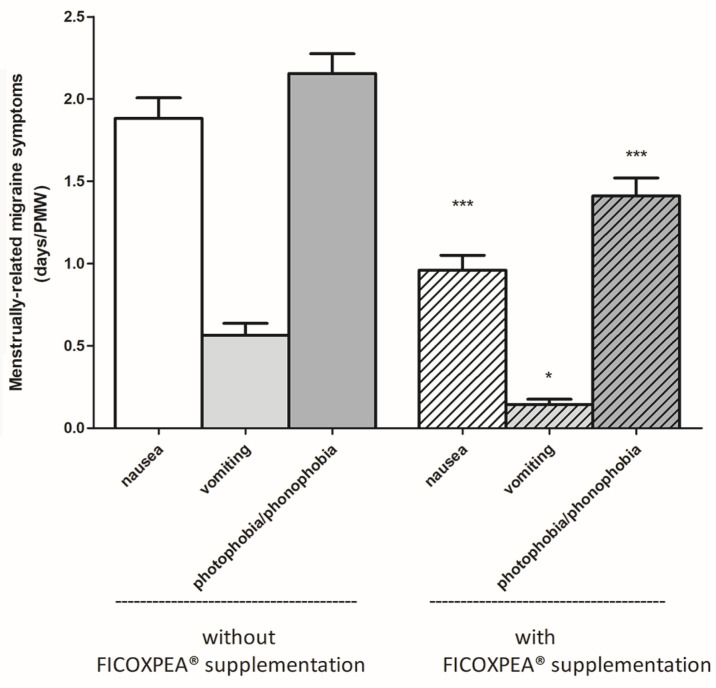
The numbers of days having menstrual migraine-associated symptoms, including nausea, vomiting, or photophobia/phonophobia during the PMW are represented as average of a three-month period of monitoring with and without FICOXPEA^®^ supplementation (n = 61). Mean values collected over three months in PMW are shown ± SEM (n = 183, <4.4% of observations missing at random). Statistical comparisons were performed using a paired *t*-test. * *p* < 0.05 and *** *p* < 0.001 compared to the same symptom in the untreated group.

The numbers of days with menstrual migraine-associated symptoms, including nausea, vomiting, or photophobia/phonophobia, during the PMW were also significantly reduced across the three monitored months of the FICOXPEA^®^ supplementation phase compared to the three months without supplementation (Figure 7). Compared with non-supplemented, each outcome decreased significantly for nausea (mean difference = −1.26, 95% CI −2.23 to −1.77, *p* < 0.001), vomiting (mean difference = −0.20, 95% CI −0.46 to −0.07, *p* < 0.01) and photophobia/phonophobia (mean difference = −1.35, 95% CI −2.41 to −1.94, *p* < 0.001). Standardized effect sizes compared to non-supplemented were: −0.72 for nausea, −0.95 for vomiting, and −0.93 for photophobia/phonophobia.

## 4. Discussion

The primary goal of acute migraine treatment is to alleviate pain and associated symptoms during an active attack. This applies to both pure menstrual migraine and menstrually related migraine, although treatment strategies may differ based on migraine severity and individual patient response. Indeed the treatment of menstrual migraine requires an individualized approach. Blocking prostaglandin production has emerged as a promising approach that aims to curtail the inflammatory component of the disorder. NSAIDs and COX-2 selective inhibitors have demonstrated effectiveness in relieving symptoms during acute attacks. Therapeutic targeting of CGRP is the most recent approach to cure migraine.

CGRP is the most important neuropeptide that is released from the trigeminal nerve endings during migraine episodes, and it is responsible for neuro-inflammation as well as modulation of nociceptive signals, not only through a direct stimulation of mast cells and smooth muscle cells but also by orchestrating a CGRP/COX-2 cascade. In fact, CGRP can enhance the expression of the COX-2 enzyme via the cAMP-PKA-CREB pathway [36,37]; the resulting elevated levels of prostaglandins (PGs) amplify the neurogenic inflammation and the nociceptive sensitization causing pain during migraine episodes. Even worse, a pathologic bidirectional stimulatory loop is created by the influence of COX-2 on CGRP release. Indeed, PGE2 increases the functionality of the Transient-Receptor-Potential-Vanilloid-1 (TRPV1) channel and causes a massive discharge of CGRP [38]. The amplified cross-linked stimulation of neuro-inflammation and CGRP-dependent vasodilatation requires an integrated therapeutic approach working on multiple targets, as single pharmacological treatments have shown limited efficacy and severe side effects. A few combinatorial therapies in migraine have been proposed recently, based on the attempt of blocking both the CGRP and COX pathways at the same time with triptan and NSAID [39] or triptan and a specific COX inhibitor [40]. Because pharmacological treatments have often demonstrated adverse reactions and pharmacokinetic interactions, addressing the neurological and inflammatory aspects of migraine could be attempted with nutraceuticals, which might have a complementary impact on reducing migraine episodes.

Among natural bioactive compounds, some may be used either as adjuncts to standard pharmacological treatments or as standalone options, depending on symptom intensity and patient preference. Phycocyanin and palmitoylethanolamide have shown particular promise owing to their anti-inflammatory, analgesic, and neuroprotective properties. Phycocyanin is a protein from the Cyanobacteria with antioxidant and anti-inflammatory properties [32] and PEA is an endogenous amide that modulates pain and inflammation by activating the peroxisome proliferator-activated receptor alpha (PPARα), a molecular pathway that suppresses the expression of pro-inflammatory factors such as nuclear factor-κB (NF-kB). Additionally, PEA exhibits pain-relieving and neuroprotective properties as an endogenously produced lipid mediator with properties similar to endocannabinoids [33]. PEA exerts inhibitory effect on sensory neuropeptide release, as demonstrated in an in vivo model of neuropathic hyperalgesia [41]. In this study, PEA, acting selectively on peripheral CB_2_-like receptors, was able to diminish plasma CGRP release evoked by the stimulation of the vanilloid subtype 1 capsaicin receptor (VR_1_/TRPV_1_). Their different mechanisms of action suggest that PC and PEA could demonstrate a complementarity in fighting neuro-inflammation. Interestingly, the synergic effect of both PC and PEA supplementation had already been tested in an in vitro model of human lung and prostate epithelial cells [42]. Indeed, Bergandi et al. demonstrated that the cotreatment protected from cytotoxicity and greatly abated both the production of radical oxygen species (ROS) and the transcription of several inflammatory cytokines. The antioxidative and anti-inflammatory properties were exerted by two main mechanisms: (1) inhibition of cyclooxygenase-2 enzyme and consequent decrease in signaling generating ROS and (2) increased synthesis of glutathione and therefore a strengthening of the natural antioxidant defenses of the cells [42]. Based on all these observations, the present study aimed at exploiting both the synergic inhibitory effects of phycocianin and PEA on COX-2 activity and the possible influence of PEA on CGRP release, with the intent of targeting both the neurological and inflammatory aspects of migraine. In this context, all patients received an oral phytotherapeutic compound containing phycocyanin and palmitoylethanolamide (FICOXPEA^®^), administered two capsules per day beginning five days before and continuing until five days after the onset of menstruation. Menstrually related migraine with aura has been included only in the most recent classification Appendix of the International Headache Society (code A1.2.0.2) [4], as in previous versions it was considered extremely unlikely for aura to occur within the PMW, which defines the period of menstrual migraine occurrence. Moreover, to meet the criteria for menstrually related migraine with aura, it remains unclear whether aura symptoms must be present during every perimenstrual window or whether an intermittent presentation is acceptable. Therefore, given the extremely low prevalence of menstrually related migraine with aura, we chose to focus exclusively on the clinically predominant form, namely migraine without aura. The data suggest potential benefit from the short-term therapy with FICOXPEA^®^ supplementation in migraine episodes occurring in PMW, the window from day −2 to day +3 relative to menstruation, because we observed both shorter attack durations and improved symptom control. In fact, as the primary outcome we discovered that the intensity of migraine was lower throughout the PMW compared to what was reported about the months preceding the supplementation. Moreover, also the monthly frequency of migraine attacks was reduced, and, more specifically, the nutraceutical mix attenuated both the frequency and the duration of attacks during the PMW. The results could be explained by the anti-inflammatory properties of the combination FICOXPEA^®^ due to the potentiated synergic inhibition of COX-2, or by the impact of COX2 inhibition on CGRP release. In fact, it has been described that cytokines can induce a COX-2 dependent pathway culminating with CGRP release in rat trigeminal ganglia neurons [43]. However, it should be noted that while this COX–CGRP involvement is plausible, it was not directly assessed in this study. Due to the enhanced anti-inflammatory activity exerted by the nutraceutical combination [42], the patients consumed fewer analgesics and the syndrome correlated with migraine during PMW was less severe in terms of nausea, vomiting, or photophobia/phonophobia.

## 5. Conclusions and Limitations

The novelty of this study lies in demonstrating a symptom relief associated with a targeted nutraceutical treatment; in fact, a short period of exposure to a mixture of PC and PEA aimed at protecting the days most critical for the onset of migraine syndrome, with double evidence: a release from pain and a reduced use of anti-inflammatory drugs.

Altogether the findings provide valuable insights and interesting observations, although the study shows some merits and flaws. Monitoring the same women over time strengthens the internal consistency of the study; however, the results should be considered with caution in consideration of certain limitations. Indeed, the retrospective design, the absence of a control group and the lack of assessment of the placebo effect limit the ability to establish a clear causality, rendering the findings preliminary. In addition, the small sample size (n = 61) and the stepwise reduction from the initial larger cohort may introduce a risk of potential selection bias, and the included patients could represent a more stable or treatment-responsive subgroup. A possible bias of baseline migraine variability was taken into account in the statistical analysis, which supported data interpretation. Instead, unmeasured parameters such as concurrent treatments and lifestyle factors were not taken into account in the confounder-adjusted analysis. In conclusion, the results of this study suggest a potential benefit of the short-term prophylaxis with phycocyanin and PEA.

## Data Availability

The data presented in this study are available within the article.

## References

[1] Macgregor E.A. (2007). Menstrual Migraine: A Clinical Review. J. Fam. Plan. Reprod. Health Care.

[2] MacGregor E.A. (2004). Oestrogen and Attacks of Migraine with and without Aura. Lancet Neurol..

[3] Wang Z., Yin Z., Wang X., Zhang Y., Xu T., Du J., Wen Y., Liao H., Zhao Y., Liang F. (2022). Brain Structural and Functional Changes during Menstrual Migraine: Relationships with Pain. Front. Mol. Neurosci..

[4] Arnold M. (2018). Headache Classification Committee of the International Headache Society (IHS) The International Classification of Headache Disorders, 3rd Edition. Cephalalgia.

[5] Welch K.M., Darnley D., Simkins R.T. (1984). The Role of Estrogen in Migraine: A Review and Hypothesis. Cephalalgia.

[6] Lipton R.B., Bigal M.E. (2005). Migraine: Epidemiology, Impact, and Risk Factors for Progression. Headache.

[7] Ceriani C.E.J., Silberstein S.D. (2023). Current and Emerging Pharmacotherapy for Menstrual Migraine: A Narrative Review. Expert Opin. Pharmacother..

[8] Caronna E., Alpuente A., Torres-Ferrus M., Pozo-Rosich P., Swanson J.W., Matharu M. (2024). Chapter 7—CGRP Monoclonal Antibodies and CGRP Receptor Antagonists (Gepants) in Migraine Prevention. Handbook of Clinical Neurology.

[9] Klan T., Gaul C., Liesering-Latta E., Both B., Held I., Hennemann S., Witthöft M. (2022). Efficacy of Cognitive-Behavioral Therapy for the Prophylaxis of Migraine in Adults: A Three-Armed Randomized Controlled Trial. Front. Neurol..

[10] Lin J.-G., Kotha P., Chen Y.-H. (2022). Understandings of Acupuncture Application and Mechanisms. Am. J. Transl. Res..

[11] Yablon L.A., Mauskop A., Vink R., Nechifor M. (2011). Magnesium in Headache. Magnesium in the Central Nervous System.

[12] Nappi R.E., Tiranini L., Sacco S., De Matteis E., De Icco R., Tassorelli C. (2022). Role of Estrogens in Menstrual Migraine. Cells.

[13] Stacey W., Bhave S., Uht R.M. (2016). Mechanisms by Which 17β-Estradiol (E2) Suppress Neuronal Cox-2 Gene Expression. PLoS ONE.

[14] Funk C.D. (2001). Prostaglandins and Leukotrienes: Advances in Eicosanoid Biology. Science.

[15] Ricciotti E., FitzGerald G.A. (2011). Prostaglandins and Inflammation. Arterioscler. Thromb. Vasc. Biol..

[16] Morgan C.T., Nkadimeng S.M. (2025). The Role of Inflammation in Migraine Headaches: A Review. FASEB BioAdv..

[17] Ailani J., Nahas S.J., Friedman D.I., Kunkel T. (2023). The Safety of Celecoxib as an Acute Treatment for Migraine: A Narrative Review. Pain Ther..

[18] Jacobs B., Dussor G. (2016). Neurovascular Contributions to Migraine: Moving beyond Vasodilation. Neuroscience.

[19] Goadsby P.J., Holland P.R., Martins-Oliveira M., Hoffmann J., Schankin C., Akerman S. (2017). Pathophysiology of Migraine: A Disorder of Sensory Processing. Physiol. Rev..

[20] Raffaelli B., Do T.P., Chaudhry B.A., Ashina M., Amin F.M., Ashina H. (2023). Menstrual Migraine Is Caused by Estrogen Withdrawal: Revisiting the Evidence. J. Headache Pain.

[21] Levy D., Moskowitz M.A. (2023). Meningeal Mechanisms and the Migraine Connection. Annu. Rev. Neurosci..

[22] Viudez-Martínez A., Torregrosa A.B., Navarrete F., García-Gutiérrez M.S. (2024). Understanding the Biological Relationship between Migraine and Depression. Biomolecules.

[23] Jenkins D.W., Feniuk W., Humphrey P.P.A. (2001). Characterization of the Prostanoid Receptor Types Involved in Mediating Calcitonin Gene-Related Peptide Release from Cultured Rat Trigeminal Neurones. Br. J. Pharmacol..

[24] Gunaydin C., Bilge S.S. (2018). Effects of Nonsteroidal Anti-Inflammatory Drugs at the Molecular Level. Eurasian J. Med..

[25] Garza I., Swanson J.W. (2006). Prophylaxis of Migraine. Neuropsychiatr. Dis. Treat..

[26] Pardutz A., Schoenen J. (2010). NSAIDs in the Acute Treatment of Migraine: A Review of Clinical and Experimental Data. Pharmaceuticals.

[27] Ong J.J.Y., De Felice M. (2018). Migraine Treatment: Current Acute Medications and Their Potential Mechanisms of Action. Neurotherapeutics.

[28] Noor N., LaChute C., Root M., Rogers J., Richard M., Varrassi G., Urits I., Viswanath O., Khater N., Kaye A.D. (2022). A Comprehensive Review of Celecoxib Oral Solution for the Acute Treatment of Migraine. Health Psychol. Res..

[29] Nickel J.C., Pontari M., Moon T., Gittelman M., Malek G., Farrington J., Pearson J., Krupa D., Bach M., Drisko J. (2003). A Randomized, Placebo Controlled, Multicenter Study to Evaluate the Safety and Efficacy of Rofecoxib in the Treatment of Chronic Nonbacterial Prostatitis. J. Urol..

[30] Abo-Elghiet F., Elosaily H., Hussein D.K., El-Shiekh R.A., A’aqoulah A., Yousef E.M., Selim H.M.R.M., El-Dessouki A.M. (2025). Bridging Gaps in Migraine Management: A Comprehensive Review of Conventional Treatments, Natural Supplements, Complementary Therapies, and Lifestyle Modifications. Pharmaceuticals.

[31] Allais G., Bussone G., De Lorenzo C., Mana O., Benedetto C. (2005). Advanced Strategies of Short-Term Prophylaxis in Menstrual Migraine: State of the Art and Prospects. Neurol. Sci..

[32] Liu R., Qin S., Li W. (2022). Phycocyanin: Anti-Inflammatory Effect and Mechanism. Biomed. Pharmacother..

[33] Alhouayek M., Muccioli G.G. (2014). Harnessing the Anti-Inflammatory Potential of Palmitoylethanolamide. Drug Discov. Today.

[34] World Medical Association (2013). World Medical Association Declaration of Helsinki: Ethical Principles for Medical Research Involving Human Subjects. JAMA.

[35] Tfelt-Hansen P., Pascual J., Ramadan N., Dahlöf C., D’Amico D., Diener H.-C., Hansen J.M., Lanteri-Minet M., Loder E., McCrory D. (2012). Guidelines for Controlled Trials of Drugs in Migraine: Third Edition. A Guide for Investigators. Cephalalgia.

[36] Anderson L.E., Seybold V.S. (2004). Calcitonin Gene-Related Peptide Regulates Gene Transcription in Primary Afferent Neurons. J. Neurochem..

[37] Lee B., Dziema H., Lee K.H., Choi Y.-S., Obrietan K. (2007). CRE-Mediated Transcription and COX-2 Expression in the Pilocarpine Model of Status Epilepticus. Neurobiol. Dis..

[38] Aikawa J., Uchida K., Takano S., Inoue G., Iwase D., Miyagi M., Mukai M., Shoji S., Sekiguchi H., Takaso M. (2018). Regulation of Calcitonin Gene-Related Peptide Expression through the COX-2/mPGES-1/PGE2 Pathway in the Infrapatellar Fat Pad in Knee Osteoarthritis. Lipids Health Dis..

[39] Brandes J.L., Kudrow D., Stark S.R., O’Carroll C.P., Adelman J.U., O’Donnell F.J., Alexander W.J., Spruill S.E., Barrett P.S., Lener S.E. (2007). Sumatriptan-Naproxen for Acute Treatment of Migraine: A Randomized Trial. JAMA.

[40] Bhatia V., Vikram V., Rattan A., Chandel A., Ashawat M.S. (2025). Neuroinflammatory Crosstalk in Migraine: Consolidated Activity of Rizatriptan and Meloxicam in Suppressing CGRP-Induced Nociception and COX-Mediated Inflammation. Inflammopharmacology.

[41] Helyes Z., Németh J., Thán M., Bölcskei K., Pintér E., Szolcsányi J. (2003). Inhibitory Effect of Anandamide on Resiniferatoxin-Induced Sensory Neuropeptide Release in Vivo and Neuropathic Hyperalgesia in the Rat. Life Sci..

[42] Bergandi L., Apprato G., Silvagno F. (2022). Antioxidant and Anti-Inflammatory Activity of Combined Phycocyanin and Palmitoylethanolamide in Human Lung and Prostate Epithelial Cells. Antioxidants.

[43] Neeb L., Hellen P., Boehnke C., Hoffmann J., Schuh-Hofer S., Dirnagl U., Reuter U. (2011). IL-1β Stimulates COX-2 Dependent PGE_2_ Synthesis and CGRP Release in Rat Trigeminal Ganglia Cells. PLoS ONE.

